# Enteropathogen co-infection in UK cats with diarrhoea

**DOI:** 10.1186/1746-6148-10-13

**Published:** 2014-01-12

**Authors:** Jasmin K Paris, Sheila Wills, Hans-Jörg Balzer, Darren J Shaw, Danièlle A Gunn-Moore

**Affiliations:** 1 Royal (Dick) School of Veterinary Studies and The Roslin Institute, The University of Edinburgh, Easter Bush Campus, Roslin EH25 9RG, Scotland; 2 Langford Veterinary Services, Langford House, Langford, Bristol BS40 5DU, England; 3 Division of IDEXX Laboratories, Vet Med Labor GmbH, 71636, Ludwigsburg, Germany

**Keywords:** Feline, Enteropathogen, Co-infection

## Abstract

**Background:**

Individual enteropathogen infections in healthy and clinically ill cats are well described, but prevalence and patterns of enteropathogen co-infection have only been reported on a limited basis. We studied enteropathogen co-infection in diarrhoeic UK cats using results of a real time PCR assay for 8 enteropathogenic species; feline coronavirus (*Co*), feline panleukopenia virus (*Pa*), *Clostridium perfringens* (*Cl*), S*almonella enterica* (*Sa*), *Giardia* spp. (*Gi*), *Tritrichomonas foetus* (*Tr*), *Cryptosporidium* spp. (*Cr*), and *Toxoplasma gondii* (*To*). Age, gender, breed and history were recorded. PCR panels from 1088 diarrhoeic cats were available for analysis.

**Results:**

Overall enteropathogen prevalence was 56.9% (*Co*), 22.1% (*Pa*), 56.6% (*Cl*), 0.8% (*Sa*), 20.6% (*Gi*), 18.8% (*Tr*), 24.4% (*Cr*) and 1.0% (*To*). Prevalence of *Co*, *Gi* and *Tr* was higher in pedigree cats compared to non-pedigree cats (DSH) and prevalence decreased with increasing age for *Co*, *Pa*, *Gi, Cr* and *Tr*. Co-infection was common: ≥2 enteropathogens were detected in 62.5% of cats, and 13.3% of cats had ≥4 enteropathogens. Mean ( $\bar{x}$) enteropathogen co-infection 2.01 (±1.3 SD), was significantly higher in pedigree cats ( $\bar{x}$ =2.51) compared to DSH ( $\bar{x}$ =1.68) and decreased with age ( $\bar{x}$ =2.64 <6 months, $\bar{x}$ =1.68 for >1 yr). More cats were negative for all 8 enteropathogens tested (12.7%) than expected. When exact combinations of co-infection were examined, *Tr* tended to be found in combinations with *Co*, *Cl,* and *Gi*.

**Conclusions:**

Multiple infections should be considered the most likely result of faecal testing in cats, and case management needs to take this into account. In contrast, the relatively high percentage of cats negative for all 8 enteropathogens tested could indicate an innate resistance to infection. Alternatively it could indicate a lack of exposure to these 8 enteropathogens or the presence of other enteropathogens not assessed by this assay.

## Background

Diarrhoea is common in domestic cats [1], and can occur as a result of gastrointestinal disease (including dietary causes, gastrointestinal infection, inflammation or neoplasia) or extra-gastrointestinal disease. A number of potential enteropathogens have been found in diarrhoeic and non-diarrhoeic feline faeces, including bacterial, viral, protozoal and other parasitic organisms [2-6]. However, reports of co-infection with 2 or more enteropathogens are surprisingly limited, and have predominantly involved *Giardia* spp. and *Tritrichomonas foetus*[7-11]. Reports of 3 or more pathogens occurring simultaneously in feline faeces are scarce. However, a recent small study examined the faeces of 50 diarrhoeic and 50 non-diarrhoeic cats entering a Florida animal shelter using a real-time PCR assay for a panel of 8 enteropathogens. Multiple organisms were identified in 44% of diarrhoeic cats in that study, but specific patterns of co-infection were not evaluated [12].

Co-infection can have clinical consequences, for example, presence of *Cryptosporidium* spp. has been associated with an increased severity of diarrhoea in *T. foetus* positive cats [13], and enteropathogen interdependence has been implicated in a study examining the effects of fenbendazole treatment in cats with concurrent *Giardia* spp. and *Cryptosporidium* spp. infection [14]. These results suggest a possible shared pathogenesis or symbiotic relationship involving selected feline enteropathogens. Evaluating enteropathogen co-infection patterns could therefore provide important information on the pathogenesis, treatment options and prognosis in affected cats.

Detection of enteropathogen infection in cats has traditionally relied upon techniques such as faecal flotation, microscopic faecal examination, antigen detection by enzyme-linked immunosorbent assays (ELISA), bacterial culture, viral isolation, immunofluorescence and electron microscopy. While these techniques are often still the most appropriate to diagnose the cause of diarrhoea in cats, they can be time consuming, costly and may require significant sample volumes, making testing for multiple enteropathogens impractical. The development of real-time PCR has enabled rapid screening of small quantities of faeces for potential enteropathogens. More recently, PCR assays capable of detecting multiple potential enteropathogens in a single faecal sample have become available for a variety of species including domestic pets [15-17]. The results generated by these assays offer for the first time the opportunity to examine co-infection patterns for selected enteropathogen species, and may form the basis for further, targeted enteropathogen testing and subsequent treatment decisions.

The primary objective of this study was therefore to identify and describe feline enteropathogen co-infection in a large population of diarrhoeic UK cats using the results obtained from the same PCR assay used in [12] for a panel of 8 enteropathogens (feline coronavirus, feline panleukopenia virus, *Clostridium perfringens*, S*almonella enterica*, *Giardia* spp., *T. foetus*, *Cryptosporidium* spp. and *Toxoplasma gondii*). Co-infection was investigated via two approaches – consideration of which enteropathogens were co-occurring in samples, and the exact combination of enteropathogens present. In addition, considering that previous reports have documented a higher prevalence of enteropathogens in juvenile cats [2,7-9,18-21] and pedigree cats [7,8,18], secondary objectives were to evaluate individual enteropathogen prevalence and frequency of co-infection in association with pedigree status and age.

The study showed that enteropathogen infection in diarrhoeic cats is common (>18% prevalence for 6 of the enteropathogens). Prevalence of feline coronavirus, *Giardia* spp. and *T. foetus* was higher in pedigree cats compared to non-pedigree cats (DSH) and decreased with age for feline coronavirus, feline panleukopenia virus, *Giardia* spp.*, Cryptosporidium* spp. and *T. foetus*. In addition, while co-infection by enteropathogens was common (62.5%), 12.7% of cats were negative for all 8 enteropathogens – which was greater than expected by chance alone. Mean ( $\bar{x}$) enteropathogen co-infection (2.01) was higher in pedigree cats ( $\bar{x}$ =2.51, DSH $\bar{x}$ =1.68) and decreased with age ( $\bar{x}$ =2.64 <6 months, $\bar{x}$ =1.68 for >1 yr). *T. foetus* was more likely to occur together with feline coronavirus, *C. perfringens* and *Giardia* spp.. Multiple infections should therefore be considered the most likely result of testing in diarrhoeic cats; however, some diarrhoeic cats may be negative for all enteropathogens tested.

## Methods

Between June 2010 and January 2012, all 1,882 feline faecal samples submitted to a reference laboratory^a^ by veterinary surgeons from first opinion small animal veterinary practices in the UK for a real-time PCR assay evaluating a panel of 8 enteropathogens^b^ were considered. However, only samples collected from diarrhoeic cats were included in the current study (N = 1151). Additional data were recorded from the submission form when available, including age, gender and breed.

Total nucleic acid was extracted from faeces by using the QIAamp DNA Blood BioRobot MDx Kit on an automated Qiagen platform (BioRobot MDx) according to the manufacturer instructions with slight modifications. Real-time PCR at IDEXX Vet Med Lab was performed using the LightCycler 480 system (Roche) with proprietary forward and reverse primers and hydrolysis probes. Target genes for enteropathogen detection using real-time PCR were as follows: feline coronavirus 7b gene (DQ010921.1), feline panleukopenia virus VP2 gene (EU252145), *Clostridium perfringens* alpha toxin gene (AM888388), *Salmonella enterica* invasion A gene (EU348366), *Giardia* small-subunit rRNA gene (DQ836339), *Tritrichomonas foetus* 5.8S rRNA gene (AF339736), *Cryptosporidium* small-subunit rRNA gene (A093489), and *Toxoplasma gondii* internal transcribed spacer-1 gene (L49390). Real-time PCR was run with 6 quality controls, including PCR-positive controls, PCR negative controls, negative extraction controls, an internal positive control (IPC) spiked into the lysis solution to monitor the nucleic acid extraction efficiency and presence or absence of inhibitory substances, RNA quality control, and an environmental contamination monitoring control. Panels containing weak or borderline positive results (n = 16) and those from pooled faecal samples (n = 47) were excluded, resulting in 1088 samples being used in this study.

### Statistical analysis

Enteropathogens were ordered for all analyses as follows: feline coronavirus (*Co*), feline panleukopenia virus (*Pa*), *C. perfringens* alpha toxin gene (*Cl*)*,* S*almonella enterica* (*Sa*), *Giardia* spp. (*Gi*), *T. foetus* (*Tr*), *Cryptosporidium* spp. (*Cr*), and *T. gondii* (*To*). Univariate general linear models with *binomial* errors were used to assess for differences in the prevalence of the 8 enteropathogen with pedigree status (DSH or pedigree) and age group (<6 months, 6–12 months and >12 months) and differences within factors were examined using standard post-hoc Tukey pairwise comparisons (*phTpc*), which adjusted for the multiple pairwise testing within a factor. A multivariable model was then run to look at the interaction between age group and pedigree status, and whether taking one factor into account resulted in a change in statistical significance associated with the other factor. Analyses of the number of enteropathogenic species detected in samples were examined in a similar way but general linear models with *Poisson* errors were used instead.

Two approaches were adopted for the analysis of co-infection. First standard chi-square (χ^2^) analyses were carried out to look at the associations of enteropathogenic species co-occurrence (hereafter termed ‘*co-occurrence’*). Here the number of observed samples with particular species was compared to what would have been expected if the species being considered were randomly distributed in samples at the frequency observed for each species on its own. Combinations of pairs, triplets etc. all the way up to all 8 species were considered. For each comparison whether other species not being considered were present or not were ignored. For example, the *co-occurrence* ‘*C:PaGi*’ indicates co-occurrence of *Pa* and *Gi* irrespective of whether the other 6 species (*Co*, *Cl*, *Sa*, *Tr*, *Cr* and *To*) were present or not.

For the second approach the exact combination of absent or present enteropathogenic species was of interest (hereafter ‘*fingerprint*’ analysis). Exact species profiles were created using results from all the 8 enteropathogenic species tested and are described according to the species present, with unlisted species being negative. For example the *fingerprint* ‘*F:CoTr*’ indicates that a faecal sample was positive for *Co* and *Tr,* and negative for all other species tested (*Pa*, *Cl*, *Sa*, *Gi*, *Cr* and *To*). These fingerprints were first examined with hierarchical cluster analyses using Ward’s minimum variance method to produce a cluster dendrogram. The significance of each particular profile was then examined by comparing the number of observed samples with that particular profile to what would have been expected to have occurred if the 8 species were randomly distributed in samples at the frequency observed for each species on its own (*i.e.* each species presence or absence was included in the estimation of what would be expected).

To take into account the likelihood of Type I errors increasing due to multiple testing, in all cases statistical significance was set as P < 0.001, and all analyses were carried out in R (version 2.15.0 © 2012 The R Foundation for Statistical Computing).

## Results

Results of 1,088 PCR panels were available where a documented history of diarrhoea was recorded. Other common clinical signs included weight loss, vomiting, anorexia, lethargy and haematochezia.

### Signalment

Available data accompanying faecal sample submissions from the cats with a history of diarrhoea included breed (100%), age (1,020 = 93.8%), gender (1,053 = 96.8%) and neuter status (952 = 87.5%). Four hundred and thirty-seven samples (40.2%) came from pedigree breeds, the remaining 651 cats were DSH. Samples were divided into 3 groups according to the age of the cat sampled, in order to expose potential differences relating to immune competence and likelihood of pathogen exposure; <6 months (236 cats, 23.1%), 6–12 months (177 cats, 17.4%), or >12 months (607 cats, 59.5%). Where recorded, there were 385 (35.4%) male neutered, 139 (12.8%) male entire, 311 (28.6%) female neutered, 117 (10.8%) female entire and 53 (4.9%) and 48 (4.4%) male and female cats respectively where neutered status was not recorded.

### Prevalence of enteropathogenic species infection

**Figure 1:**
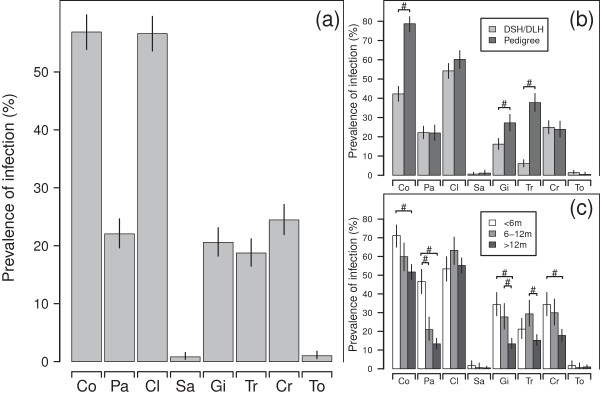
**Diarrhoeic cat individual enteropathogenic species prevalence.** Bar plots of prevalence of infection of individual enteropathogens in cats with a documented history of diarrhoea (feline coronavirus (*Co*), feline panleukopenia virus (*Pa*), *Clostridium perfringens* alpha toxin gene (*Cl*)*,* S*almonella enterica* (*Sa*), *Giardia spp*. (*Gi*), *Tritrichomonas foetus* (*Tr*), *Cryptosporidium spp*. (*Cr*), and *Toxoplasma gondii* (*To*)) detected in feline faecal samples by Real-time PCR. **(a)** All samples; grouped according to **(b)** pedigree status or **(c)** age group. #P < 0.001. Vertical black lines represent 95% exact binomial confidence intervals.

The overall prevalences of enteropathogenic species were 56.9% feline coronavirus, 22.1% feline panleukopenia virus, 56.6% *C. perfringens*, 0.8% *S. enterica*, 20.6% *Giardia* spp., 18.8% *T. foetus*, 24.4% *Cryptosporidium* spp. and 1.0% *T. gondii* (Figure 1a).

Faecal samples from pedigree cats were significantly more likely than DSH to be positive for feline coronavirus (78.7% vs. 42.2%), *Giardia* spp. (27.2% vs. 16.1%) and *T. foetus* (37.8% vs. 6.0%, P < 0.001), with no such differences for the other 5 species (P > 0.051, Figure 1b). There were also significant differences in the prevalence of infection between the three age groups for feline coronavirus, feline panleukopenia virus, *Giardia* spp., *T. foetus* and *Cryptosporidium* spp. (P < 0.001, P > 0.091 for the other 3 species, Figure 1c). In particular, young cats (<6 months) had significantly higher prevalences of infection than both the older cat groups for feline panleukopenia virus (46.6% *vs.* 20.9% (6-12 m) and 13.3% (>12 m), P < 0.001). In addition, the youngest cats also had significantly higher prevalence of infection than the oldest cats for 3 other species (*Co* 71.2% *vs.* 51.7%, *Gi* 34.3% *vs.* 13.3% and *Cr* 34.3% *vs.* 17.8%, P < 0.001). Furthermore, 6–12 month old cats had significantly higher prevalence of infection compared to the oldest cats for 2 species (*Gi* 27.7% *vs.* 13.3%; *Tr* 29.4% *vs.* 15.2%, P < 0.001, Figure 1c). There was no statistically significant interaction between any species found in a sample and age and pedigree status (P > 0.063). Taking age into account made no qualitative differences to the univariate pedigree results, with differences still observed for feline coronavirus, *Giardia* spp. and *T. foetus* (P < 0.001) and no other differences (P > 0.125). A similar lack of qualitative change in significance shown in Figure 1c was observed for age groups taking into account pedigree status for all but 1 of the enteropathogens, with the difference between 6-12 m and >12 m no longer different for *T. foetus* (P = 0.022).

### *Co-occurrence* analysis

**Figure 2:**
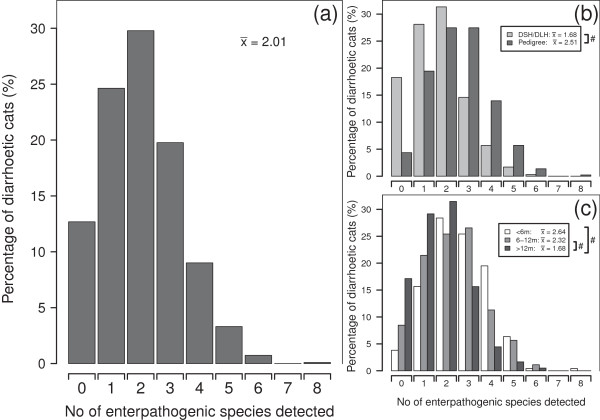
**Number of enteropathogenic species found in diarrhoeic cats.** Bar plots of percentage of faecal samples as a function of the number of enteropathogenic species detected by real-time PCR in **(a)** samples from cats with a documented history of diarrhoea; **(b)** grouped according to pedigree status or **(c)** age group. #P < 0.001, $\bar{x}$ = arithmetic mean.

Enteropathogenic species co-infection was a common finding, with 62.5% of the 1,088 samples having 2 or more species (mean $\bar{x}$ =2.01 ± 1.3SD, Figure 2a), and 13.3% with 4 or more species detected. However, 12.7% of the samples submitted were negative for all 8 species. No samples had 7 species present but 1 sample did have all 8. Mean species carriage in pedigree cats ( $\bar{x}$ =2.51 ± 1.3) was significantly higher than in DSH cats ( $\bar{x}$ =1.68 ± 1.2) (P < 0.001, Figure 2b), with 86.2% of the cats with none of the 8 species detected being DSH. Furthermore, there were differences in mean carriage with age group, with cats >12 months old ( $\bar{x}$ =1.68 ± 1.2) having significantly fewer species per sample compared to <6 months old ( $\bar{x}$ =2.64 ± 1.3, P < 0.001) and 6–12 months old cats ( $\bar{x}$ =2.32 ± 1.4, P < 0.001, Figure 2c). In addition, 81.3% of cats with none of the 8 species detected were >12 months old and 68.8% of were DSH cats >12 m old. The differences observed in age groups remained when differences in pedigree status were first taken into account, and *vice versa* (P < 0.001).

**Figure 3:**
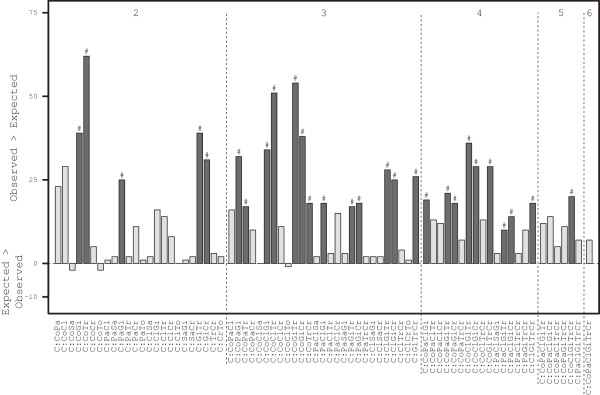
**The difference between observed and expected co-occurrence of 2 or more of the 8 enteropathogenic species in diarrhoeic cats.** Bar plot of the difference in *co-occurrence* of 2 or more of the 8 enteropathogenic species in samples from cats with a documented history of diarrhoea, irrespective of what other species were present, compared to what would be expected as a product of each species’ individual occurrence. Only those *co-occurrence* combinations where either the number expected or observed was ≥3 are shown (37/219 (16.9%) possible co-occurrence combinations). The *co-occurrence* results are ordered left to right according to the number of species, in the order *Co, Pa, Cl, Sa, Gi, Tr, Cr* and *To*. The dark bars indicate statistically significant difference in observed minus expected with #P < 0.001. Numbers at the top of the graph indicate the number of enteropathogenic species.

The analysis of *co-occurrence* data found that 28 of the possible co-occurrences were present more than would be expected if enteropathogens were present in samples at random (P < 0.001, Figure 3). Only 4 of the possible co-occurrences were observed less than expected, but differences were ≤2 (P > 0.472). Pair-wise consideration revealed feline coronavirus co-occurred with *Giardia* spp., and *T. foetus,* more frequently than expected, but not with *C. perfringens* (despite *C:CoCl* being the most common co-occurrence – 379 samples, P = 0.092) or feline panleukopenia virus. In contrast, there was greater co-occurrence of feline panleukopenia virus and *Giardia* spp., and *Giardia* spp. with both *Cryptosporidium* spp. and *T. foetus,* respectively, Figure 3). There were significantly greater co-occurrence in 13/56 (23%) of 3-way co-occurrences, 9/70 (13%) of 4-way co-occurrences and 1/56 (2%) of 5-way co-occurrences (Figure 3). No one enteropathogenic species dominated these 23 combinations – with 6 of the 8 species occurring with similar frequencies in the 23 combinations: feline coronavirus (14/23), feline panleukopenia virus (10), *C. perfringens* (12), *Giardia* spp. (20), *T. foetus* (13) and *Cryptosporidium* spp. (11), reflecting the 6 most common enteropathogens observed (Figure 1a).

### *Fingerprint* analysis

**Figure 4:**
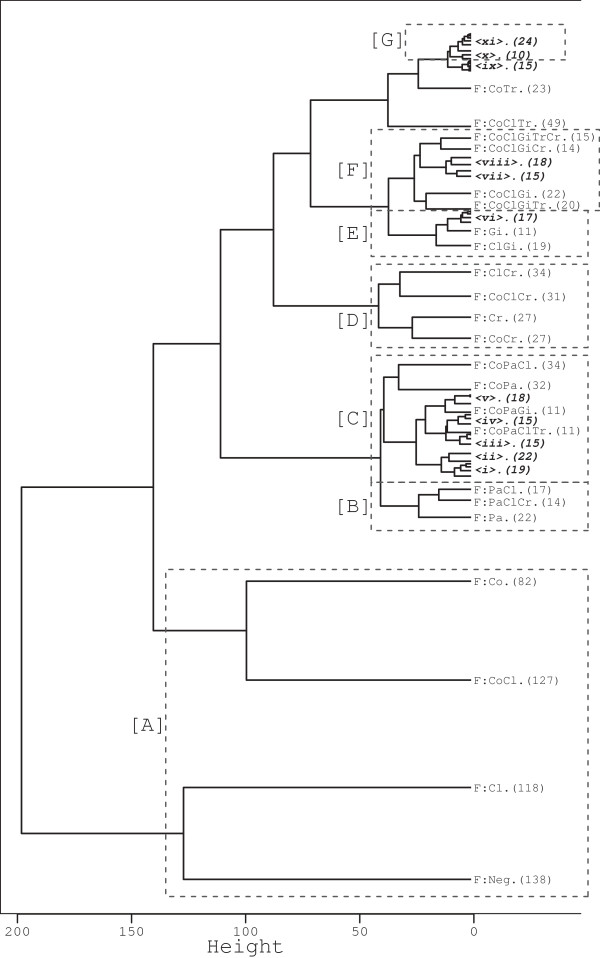
**Hierarchical cluster dendrogram of enteropathogenic species *fingerprints *from diarrhoeic cats.** Hierarchical cluster dendrogram of enteropathogen *fingerprints* using Ward’s minimum variance method of enteropathogenic species presence detected in feline faeces from cats with a documented history of diarrhoea. The number in round brackets is the number of samples with that *fingerprint*. **[A]**-**[G]** and associated dotted polygons - sample/clusters referred to in text. *Fingerprints*: ***<i>*** *F:PaCr* (7), *F:CoPaCr* (5), *F:CoPaGiTrCr* (4), *F:CoPaGiCr* (3); ***<ii>*** *F:CoPaClGiCr* (10), *F:CoPaClCr* (12); ***<iii>*** *F:CoPaClGiTrCr* (7), *F:CoPaClGiTr* (6), *F:CoPaClSaGiTrCrTo* (1), *F:CoPaClSaGiTr* (1); ***<iv>*** *F:CoPaGiTr* (7), *F:CoPaTr* (5), *F:PaTr* (3); ***<v>*** *F:CoPaClGi* (10), *F:PaClSaGi* (1); *F:PaClGi* (7); ***<vi>*** *F:GiCr* (6), *F:ClGiCr* (5), *F:PaClGiCr* (3), *F:PaGi* (1), *F:PaGiCr* (2); ***<vii>*** *F:CoGiCr* (7), *F:CoGiTrCr* (8); ***<viii>*** *F:CoGiTr* (9), *F:CoGi* (11); ***<ix>*** *F:ClSaCr* (2), *F:CoClSa* (1), *F:ClSa* (1), *F:Sa* (1), *F:PaSaCrTo* (1), *F:CrTo* (1), *F:CoClCrTo* (1), *F:ClCrTo* (1), *F:CoTo* (1), *F:To* (2), *F:CoClTrTo* (1), *F:PaClTo* (1), *F:ClTo* (1); ***<x>*** *F:CoClTrCr* (5), *F:CoTrCr* (5); and ***<xi>*** *F:ClTr* (8), *F:Tr* (5), *F:TrCr* (4), *F:ClGiTrCr* (1), *F:GiTr* (1), *F:ClGiTr* (1), *F:ClTrCr* (2), *F:PaClTr* (1), *F:CoPaClTrCr* (1).

**Figure 5:**
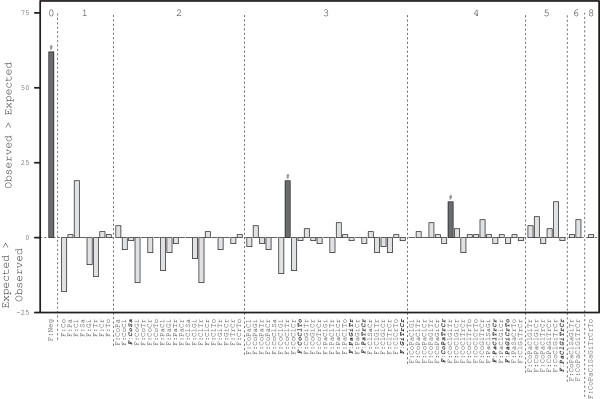
**Observed minus expected occurrence of enteropathogenic species *fingerprints *in diarrhoeic cats.** Bar plot of *fingerprint* profiles of the difference in the observed occurrence of each *fingerprint* as detected in feline faeces from cats with a documented history of diarrhoea compared to the expected occurrence. *Fingerprint* profiles are coded according to exact enteropathogenic species present. There were 72 profiles observed + 9 profiles (***bold italics***) that were expected to be observed but were not. The profiles are ordered left to right according to their number of species in the order *Co, Pa, Cl, Sa, Gi, Tr, Cr* and *To*. The dark bars indicate statistically significant difference in observed minus expected with #P < 0.001.

After evaluating co-occurrence, analysis was carried out to evaluate the exact combination of enteropathogens (fingerprint) present or absent in samples. Out of a possible 256 faecal profiles, only 72 (28%) were observed (Figure 4). The most striking result from the dendrogram is that 43% of all samples were clustered in 4 profiles consisting of the 12.7% samples which were not positive for any of the 8 species and 327 (30.0%) samples in which either just feline coronavirus (82) or *C. perfringens* (118) was detected or both (127, Figure 4[A]), with none of the other species detected. However, in terms of statistical significance the profiles of *F:Co*, *F:Cl* and *F:CoCl* did not occur more than would be expected by chance given the overall prevalences of infection of the 8 enteropathogenic species (P > 0.052, Figure 5). In contrast, there were statistically more *F:Neg* profiles (138) than would be expected (76, P < 0.001).

The next most common fingerprint was *F:CoClTr* (49, Figure 4), which was observed significantly more than expected (P = 0.001, Figure 5). The remaining 574 samples (excluding 23 *F:CoTr* samples, and 15 samples which represented 13 profiles (Figure 4*<ix>*), including the *F:Sa* (N = 1) and *F:To* (N = 2) mono infections) could be allocated into 6 broad clusters representing 53 other observed profiles (Figure 4). There was a cluster of feline panleukopenia virus with *Cryptosporidium* spp. and/or *C. perfringens* (N = 53, Figure 4[B]) but levels were as expected for *F:Pa, F:PaClCr* and *F:PaCl* (P > 0.044, Figure 5). There was also another very broad cluster of feline coronavirus with feline panleukopenia virus and other enteropathogenic species (N = 177, Figure 4[C]) with *F:CoPaCl* and *F:CoPa* representing 37% of that cluster, though these profiles were not observed more or less than expected (P > 0.091, Figure 5). There was another apparent cluster of *Cryptosporidium* spp. with feline coronavirus and/or *C. perfringens* (N = 119, Figure 4[D]), and a small cluster of *Giardia* spp. and *C. perfringens* (N = 47, Figure 4[E]), though none of the profiles in clusters [D] and [E] were present more or less than expected (P > 0.089, Figure 5).

The next cluster consisted of feline coronavirus with *Giardia* spp. and other species (N = 106, Figure 4[F]). However, in contrast to clusters [B]-[E], one profile was not as expected: *F:CoClGiTr* (N = 20) occurred more than expected (P < 0.001, Figure 5). There was a final small cluster of low numbers of samples mainly consisting of *T. foetus* with *Cryptosporidium* spp. and/or *C. perfringens* and other species (N = 34, Figure 4[G]).

There were an additional 9 fingerprints that were expected to occur given the relative occurrence of individual enteropathogenic species where none were observed (*F:CoSa*, *F:CoClTo*, *F:PaGiTr*, *F:PaTrCr*, *F:GiTrCr*, *F:CoPaTrCr*, *F:PaClTrCr*, *F:PaGiCrTo* and *F:PaClGiTrCr*, Figure 5). However, these were each only expected to be observed in 2 or fewer samples. This left 175 potential profiles not observed and these profiles all contained the 2 enteropathogenic species that occurred at very low (≤1%) frequencies (*Sa* and *To*). If these 2 species were excluded, then there were 64 possible fingerprints with the 6 other species and of these 56 (88%) fingerprint profiles were observed (Figure 4), with 6 of the 9 profiles described above expected but not observed.

## Discussion

This is the first large scale study to evaluate enteropathogenic species co-infection patterns in cats. Several methods were used to evaluate co-infection. A *co-occurrence* analysis was used to look at co-infection with 2–8 species, irrespective of the presence or absence of the other enteropathogens. This permitted evaluation of which of the 8 enteropathogenic species were likely to occur in diarrhoeic cat samples submitted by first opinion veterinarians as determined by this type of diagnostic assay. However, the *co-occurrence* approach did not evaluate enteropathogen absence as a determinant of precise co-infection patterns. Co-infection patterns were therefore also evaluated using *fingerprint* analysis, which took into account the absence or presence of each of the 8 enteropathogens studied. This permitted consideration of the interaction between specific enteropathogens. This study demonstrated that multiple co-infections were common, with at least 2 of the 8 enteropathogens detected in 62.5% cats. Moreover, the results indicated that co-infection was often not a random event, in terms of which of the 8 enteropathogens were observed to occur together. Another interesting result was that 12.7% of cats with reported diarrhoea had none of the 8 enteropathogen species tested by this assay, which was significantly higher than would be expected based on chance alone.

In this study, the prevalence of individual enteropathogens (Figure 1a) correlated well with previous reports. Feline coronavirus was identified in 56.9% of diarrhoeic faecal samples, consistent with previous reports of 41-75% [22,23]. Feline panleukopenia virus was detected in 22.1% of samples, which is comparable to a detection rate of 19.2% in 52 faecal samples from cats with diarrhoea by electron microscopy in a previous study [24], but higher than the anecdotal incidence of clinical feline panleukopenia infection in the UK. Possible explanations include asymptomatic infection, passive viral carriage, or false positive results occurring as a result of modified live vaccine administration within the preceding 2 weeks [25]. Alternatively, PCR cross reactivity with canine parvovirus (CPV) could be responsible, given that CPV was detected by PCR in 37% of faecal samples from asymptomatic shelter cats in a recent study [26]. The prevalence of *C. perfringens* alpha toxin gene was 56.6%, consistent with previous data [5,6]. *Giardia* spp. were detected in 20.6% samples, corresponding to previous prevalence estimates ranging from 0.58-80% depending on the test population and detection method employed [19,20,27-29]. *T. foetus* was identified in 18.8% of samples, consistent with previous prevalence data of 14.4-82% [8,18,27]. The prevalence of *Cryptosporidium* spp. in this study was 24.4%, which is greater than the 2–12.3% reported previously [6,28,30]. The discrepancy might reflect improved sensitivity of the PCR assay for detection of *Cryptosporidium* spp. compared with traditional faecal evaluation and immunoassay methods [28]. As in previous studies, *T. gondii*[6] and S*almonella enterica*[4,6] were detected infrequently, with prevalence values of 1.0% and 0.8% respectively.

In addition to showing that the 8 enteropathogenic infections occur frequently individually, this study also identified frequent enteropathogen co-infection. Using an 8-way PCR assay, co-infection with ≥ 2 enteropathogenic species was observed in 62.5% faecal samples, and overall mean species carriage was 2.01 (Figure 2a). A higher mean enteropathogen carriage was identified in both pedigree (Figure 2b) and young cats (Figure 2c), consistent with the higher prevalence of both *Giardia* spp. and *T. foetus* observed in these groups and also the higher prevalence of feline coronavirus, feline panleukopenia virus and *Cryptosporidium* spp. in the youngest cats in this study and in previous reports [2,7-9,18,20]. Consistent with these observations, 69% of cats with no enteropathogens detected were found to be DSH cats > 12 months of age. The increased enteropathogen carriage in pedigree and young cats could reflect genetic or age related reductions in immune-competence, or increased contact with other cats in cattery, breeding, and cat-show establishments. Increased housing density has been identified as a risk factor for *T. foetus*[27], feline coronavirus [31-33] and *Giardia* spp. infections in cats [34], possibly reflecting the common faeco-oral route of infection. Alternatively, housing conditions may influence disease risk in cats as a result of stress [35]. In the case of gastrointestinal disease, the mechanism responsible is thought to involve alterations in epithelial barrier function [36].

Previous reports of feline enteropathogen co-infection have focused predominantly on infections observed along with *T. foetus*. Several studies have described co-infection with *T. foetus* and *Giardia* spp., with co-infection prevalence rates ranging from 4.3% to 54% [7,8,27]. In a study of experimentally-induced *T. foetus* infection in cats, the duration and severity of diarrhoea was significantly greater in cats with chronic pre-existing and asymptomatic *C. parvum* infection than in specific pathogen free cats. In addition, co-infected cats were more likely to suffer episodes of acute self-limiting diarrhoea in the chronic phase (> 7 weeks) of *T. foetus* infection, particularly following diagnostic procedures or changes in antibiotic therapy [13]. The authors of that study concluded that fluctuations in the intestinal microbiota may be necessary to produce the clinical manifestations of *T. foetus* infection. The intimate relationship between *T. foetus* and the intestinal microbiota is consistent with the observation that trichomonads are obligate parasites dependent on endogenous bacterial flora and host secretions for acquisition of essential nutrients [37]. The results from the current study’s fingerprint analysis are consistent with this observation: *T. foetus* was observed more commonly than expected as a fingerprint and co-associated with feline coronavirus and *C. perfringens*, with the potential addition of *Giardia* spp.

Statistically, the most significant fingerprint analysis result indicated that a much greater proportion of faecal samples were negative for all 8 of the enteropathogenic species than expected (Observed 138, Expected 76, Figure 5); this corresponded to an overall percentage of 12.7% of the cats. Since all the cats had documented diarrhoea on sample submission, this finding may indicate that some cats are intrinsically resistant to infection by these 8 enteropathogenic species, but have diarrhoea caused by another mechanism. Alternatively, some cats may not have been exposed to these 8 species, and may have been infected with enteropathogenic species not assessed by this diagnostic panel (for example *Campylobacter* spp. or *Isospora* spp.). Further studies will be required to identify potential common factors in this group of cats. However, it is of interest to note that 69% of the diarrhoeic cats with none of the 8 species detected were >12 months old DSH cats. This is consistent with our finding of smaller numbers of enteropathogen species in older DSH cats.

While fingerprint analysis was used to examine the exact combinations of the 8 species present in the faecal samples, the association between individual sets of species was also examined separately, irrespective of the presence or absence of any enteropathogens not currently under consideration (*co-occurrence* analysis). Feline coronavirus was identified more frequently than expected together with *Giardia* spp., and *T. foetus* in this study*.* Previous work has shown that a proportion of cats become chronic carriers following infection with feline coronavirus [38-40]. These cats shed the same strain of coronavirus for years [38], suggesting this enteropathogen has developed mechanisms to suppress the host immune response, such as induction of TNFα release by infected cells and subsequent lymphocyte apoptosis [41,42]. A reduction in local host immune responses within the intestinal tract may explain the increased frequency of feline coronavirus co-occurrence with other enteropathogens.

Significant co-occurrence was also identified for *Giardia* spp*.* with both *Cryptosporidium* spp*.* and *T. foetus,* potentially reflecting shared protozoal features. Similarities between *T. foetus* and *Giardia* spp. have been identified at the molecular level, including molecular and genetic traits, suggesting that they are of sister lineages [43]. Pathogenic mechanisms are reported to be similar for *Cryptosporidium* spp. and *Giardia* spp., with common features including malabsorption, hypersecretion and disrupted epithelial barrier function [44,45]. Considering that pathogenic mechanisms proposed for *T. foetus* include alterations in the normal flora, adherence to the epithelium, and elaboration of cytokines and enzymes [46], infection with *Giardia* spp. and *Cryptosporidium* spp*.* could predispose to *T. foetus* infection.

There were several limitations to this study. Given the retrospective nature of the study, there was no control over the data provided at the time of sample submission, and no follow up in terms of treatment and outcome. Of the samples submitted, 35% were not accompanied by any historical information, and in the remaining 65% it was not possible to rule out clinical signs that were not mentioned on the submission form. For this reason, only cats with a documented history of diarrhoea were included. The lack of background information (for example: housing, diet, number of cats in household, geographical location, show attendance) prevented evaluation of additional risk factors for individual and multiple enteropathogen infection. In addition, the effect of prior treatment (antimicrobials, anthelmintics) on prevalence and patterns of enteropathogen co-infection could not be established. In the absence of a healthy control population, it was not possible to determine the clinical significance of individual or multiple enteropathogen infections in cats. Future prospective studies which include a control population of healthy cats are therefore required.

This study used PCR to investigate the presence of 8 enteropathogenic species and explore their co-carriage. It is important to note that even though an organism is detected by PCR, it does not mean it is necessarily the cause of the documented diarrhoea. Furthermore, the highly sensitive aspect of PCR assays may result in positive results in the presence of negligible enteropathogen burdens. PCR has been accepted as the gold standard technique for diagnosis of *T. foetus* infection in cats [27,47], but alternative diagnostic techniques may be preferred for other enteropathogen infections. Another approach to aid diagnosis of the cause of the diarrhoea would be to combine conventional faecal testing (using microscopy for parasitology, and bacterial faecal culture and toxin testing) with a modified PCR panel to document *C perfringens* alpha toxin gene (quantitative assay), *C. perfringens* enterotoxin gene (quantitative assay), plus *C. coli*, and *C. jejuni*.

## Conclusions

This is the first large scale study examining co-infection patterns of 8 enteropathogenic species in diarrhoeic cats. The results show that co-infection by these species is common in cats, and that specific patterns of co-infection occur both more and less commonly than expected, indicating that infection by different species is not a random process but clustered. *T. foetus* is more likely to occur together with feline coronavirus, *C. perfringens*, and *Giardia* spp. in diarrhoeic feline faeces. Finally, the proportion of cats with none of the tested enteropathogenic species detected is greater than would be expected based on chance alone, suggesting that some cats may have an intrinsic resistance to infection to these species, or a lack of environmental exposure specific to these species. Further work is required to establish whether different patterns would be observed in healthy cats and the relationship between the causes of diarrhoea and the detection of one or more enteropathogens in faeces.

### Endnotes

^a^IDEXX Laboratories Limited, Grange House, Sandbeck Way, Wetherby, West Yorkshire, LS22 7DN, United Kingdom.

^b^Feline Diarrhoea RealPCR**™** Panel.

## Competing interests

JB works for IDEXX Laboratories which offers the 8-way feline PCR assay as a commercial test.

## Authors’ contributions

JB carried out PCR testing, SW retrieved patient signalment, history (where available) and PCR data from stored records, DJS participated in the design of the study, performed the statistical analysis and helped to draft the manuscript, JP and DGM conceived of the study, participated in its design and co-ordination and helped draft to the manuscript. All authors read and approved the final manuscript.
